# Tongue Sucking as a Cause of the Protrusion of the Incisor Teeth

**Published:** 1882-02

**Authors:** Appleby King

**Affiliations:** Worcester.


### ARTICLE V.

### *On Tongue Sucking as a Cause of Protrusion of the Incisors.*

BY APPLEBY KING, L. D. S. I., WORCESTER.

The ordinary causes of protrusion of the upper incisors are well known, but there is one cause of the deformity which, so far as I know, has not yet been brought under the notice of the profession. The connection between the two phenomena, protruding incisors and the habit of tongue sucking, was forced upon my attention quite by accident, under circumstances which I will briefly relate. The following cases have all been under my immediate care; their etiology could not be traced to any of the generally known causes, and I think I will be able to show that they were totally due to "tongue sucking." I adopt this expression because it was the term used by the patient who first introduced the habit to me.

Some fourteen years since, Mrs. S—, of Shrewsbury, consulted me respecting her very much disfigured mouth. The roots of the teeth were exposed about the twentieth of an inch out of the gums, showing a very strong partiality to be in a line with the nose. The patient informed me that a denitst had told her he could force them into their proper position, and she wished me to perform this operation.

My patient having reached the very interesting age of forty-five, I advised her not to entertain any hope of success even if any one should attempt so absurd a treatment. Eventually consent was given for the removal of the four front teeth, which, being sound and of good color, were reinserted on a gold plate. In making a careful examination of this mouth, I found nothing satisfactory to determine what was the cause of the deformity. After asking several questions, amongst others, I inquired whether she ever had a swollen tongue, and to my great astonishment, she informed me that she had for some years been in the habit of "sucking her tongue," and it was her opinion that the pressure of that organ on the teeth had been the cause of the mischief. There was no mistaking this fact, for upon asking her to perform the operation (though she had abandoned the habit for some years, she had not forgotten the muscular action) the tongue was brought upon the lingual surfaces of the teeth with great force ; the action being just that of an infant, but instead of the teat being present to press into the palate, the tongue otherwise directed its pressure on the four front teeth. If the mouth-piece of a feeding bottle be removed from the mouth of an infant while sleeping the exact process will be witnessed.

This case I must say rather startled me, as I had never heard of the habit before, and since that time I have been on the look-out for other cases, the result of my observation being that there are two forms of tongue sucking, a flat spreading action of the tongue, involving all the four upper incisors, and a pointed pressure when the tongue is screwed to a point and its force directed to the upper central teeth only.

In June, 1869, Sarah T——, of Kidderminster, came with severe tooth-ache ; I found the four upper incisors slightly protruding, a large space between the two central teeth, and slight spaces between the laterals and canines ; rather a large, well-developed jaw, comparatively good teeth, general condition healthy. On my putting the question, Do you suck your tongue? the reply was, "I do not understand your meaning." Feeling convinced in my own mind that it was one of those cases, I determined to follow it up. On seeing the patient's mother, and informing her of my suspicions with regard to this habit, she at once admitted "that her daughter was always at it," and wished she could break her of it. On my undertaking to do this, and assuring my patient that I could reduce the deformity, she readily consented, offering every assistance, which greatly facilitated success.

The treatment in this case was as follows:

A vulcanite plate was made covering the whole of the palate and upper molar teeth. A shield having been made in front of the lingual surfaces of the four upper incisors, a stout metal band was carried from one buccal side of the superior maxilla to the other, close to the projecting labial points of the four front teeth. The lower molars and bicuspids articulated on the vulcanite plate, pressure was then obtained by forcing lint between the metal band and the projecting points of the teeth. This the patient managed most successfully herself. When the sucking commenced, the teeth were protected by the shield curling up over the lingual surfaces of the four front teeth. After the teeth were brought into their proper position, much difficulty arose in getting the patient to abandon the old habit, for, though willing to give it up, the force of habit was too strong and she indulged in it without being conscious of the fact. This trouble, however, was overcome by inserting pegs in the guard and changing their position each day. The tongue discovering the altered position of the pegs was the signal to desist. Case completed in two years.

The late Mr. John Ailman, Senior Surgeon to the Kidderminster Infirmary, brought me an out-patient, a girl of 13, who sucked both thumb and tongue. The sucking in this case was pointed, *i. e.* only the tip of the tongue came in contact with the two central teeth. The thumb had been placed in the centre of the front teeth, and they were forced quite out of position. I treated this case for a few months and lost sight of it through the patient leaving the district.

I have frequently inquired of my professional brethren if they had met with any of these cases, and I have never yet found any one who had followed one up, though some admitted they had met with cases of tongue sucking.

In a report of proceedings of the Odontological Society, May 15th, 1881, Mr. Henry Sewill mentioned a case which looks to me like one of tongue sucking. Speaking of protrusion of the upper teeth, he exhibited models of a case, and observed that from his experience the disease occurred only in women, and was not noticeable before the age of twenty-five; the teeth then began to protrude but did not become loose until quite a late stage of the disease. There was no discharge from the gums, no inflammation, no wasting of the alveoli, and no similar disease of the other teeth, and he could not account for the deformity. No remark was made upon the case, with the exception of some one falling back to thumb sucking.

It is not easy to find these cases out, for I have known patients who after denying they have indulged in this abuse commence it the moment one's back is turned. Again you have to be most careful in mentioning the subject, for if you ask a young lady, "Do you suck your tongue?" the reply will immediatly be with an embarrassed air, "No!"

I feel sure that the habit exists to a far greater extent than is generally believed. In most cases the patient will come under observation when the injury is done, and too late to do any good. Young patients are the last to come under treatment, because there is little inconvenience to themselves, though the mischief may be great in effect. In conclusion, it must not be understood that I am trying to lay it down as a demonstrable fact that every girl found sucking the tongue must have this deformity, or that every patient with a V-shaped jaw or protruding upper teeth must have necessarily given way at some time to the abuse I have mentioned. I have not, however, the least hesitation in saying that this is one of the causes of the deformity, and when it occurs in a limited degree, where there is a disposition to congenital weakness, the mischief is great, and the same injury may result under healthy conditions where the habit is persistent and long continued.—*British Journal of Dental Science.*

